# Iron -Doped Mesoporous Nano-Sludge Biochar via Ball Milling for 3D Electro-Fenton Degradation of Brewery Wastewater

**DOI:** 10.3390/nano15191530

**Published:** 2025-10-07

**Authors:** Ju Guo, Wei Liu, Tianzhu Shi, Wei Shi, Fuyong Wu, Yi Xie

**Affiliations:** School of Brewing Engineering, Moutai Institute, Renhuai 564507, China; guoju@mtxy.edu.cn (J.G.); shitianzhu@mtxy.edu.cn (T.S.);

**Keywords:** iron-doped modification, sludge biochar, three-dimensional electro-Fenton technology, organic wastewater from sauce-flavor liquor brewing

## Abstract

To address the challenges of complex composition, high chemical oxygen demand (COD) content, and the difficulty of treating organic wastewater from brewery wastewater, as well as the limitations of traditional Fenton technology, including low catalytic activity and high material costs, this study proposes the use of biochemical sludge as a raw material. Coupled with iron salt activation and mechanical ball milling technology, a low-cost, high-performance iron-doped mesoporous nano-sludge biochar material is prepared. This material was employed as a particle electrode to construct a three-dimensional electro-Fenton system for the degradation of organic wastewater from sauce-flavor liquor brewing. The results demonstrate that the sludge-based biochar produced through this approach possesses a mesoporous structure, with an average particle size of 187 nm, a specific surface area of 386.28 m^2^/g, and an average pore size of 4.635 nm. Iron is present in the material as multivalent iron ions, which provide more electrochemical reaction sites. Utilizing response surface methodology, the optimized treatment process achieves a maximum COD degradation rate of 71.12%. Compared to the control sample, the average particle size decreases from 287 μm to 187 nm, the specific surface area increases from 44.89 m^2^/g to 386.28 m^2^/g, and the COD degradation rate improves by 61.1%. Preliminary investigations suggest that the iron valence cycle (Fe^2+^/Fe^3+^) and the mass transfer enhancement effect of the mesoporous nano-structure are keys to efficient degradation. The Fe-O-Si structure enhances material stability, with a degradation capacity retention rate of 88.74% after 30 cycles of use. When used as a particle electrode to construct a three-dimensional electro-Fenton system, this material demonstrates highly efficiency in organic matter degradation and shows promising potential for application in the treatment of organic wastewater from sauce-flavor liquor brewing.

## 1. Introduction

The organic wastewater generated from the brewing of sauce-flavor liquor (hereinafter referred to as “brewing wastewater”) has a complex composition, containing a variety of organic compounds such as alcohols, aldehydes, acids, and esters. It is characterized by high chroma, a high content of suspended solids, and is classified as high-concentration, refractory organic wastewater. This limits the sustainable and high-quality development of the sauce-flavor liquor industry [1,2].

Traditional treatment methods primarily include physical, chemical, and biochemical processes. Physical processes separate suspended pollutants through various physical means (e.g., adsorption, filtration, centrifugation) without altering the chemical properties of the substances involved [3,4]. However, these methods are often inefficient and cannot consistently meet discharge standards, making them mostly suitable as a pretreatment step. Chemical processes, such as oxidation, decomposition, and neutralization-precipitation, remove pollutants through chemical reactions [5,6,7]. Although these methods are well-established, with stable treatment effects and broad applicability, they suffer from limitations including low removal efficiency and high treatment costs. Biochemical processes utilize the metabolic activities and adsorption capabilities of microorganisms to enrich, degrade, and remove pollutants. These processes are widely adopted in the liquor industry [1,8]. However, they are also associated with challenges such as the generation of biochemical sludge solid waste, the emission of toxic and harmful gases, high equipment costs, and significant land use. Therefore, the development of an efficient, stable, and cost-effective treatment technology for brewing wastewater has become a critical goal for researchers and industry professionals.

The three-dimensional (3D) electro-Fenton process is a type of advanced oxidation technology [9,10,11]. Compared with the traditional two-dimensional (2D) system, the 3D electro-Fenton system introduces particle electrodes, resulting in enhanced mass transfer rates, enlarged specific surface area, and improved H_2_O_2_ generation efficiency. These advantages significantly promote the oxidation of organic pollutants and enhance the overall treatment performance of wastewater. Consequently, the 3D electro-Fenton process has been widely applied in various industrial wastewater treatments, including dairy, printing and dyeing, and coal chemical sectors [12,13,14]. For example, Mohammad et al. reported that the electro-Fenton method achieved a 95.8% removal of COD and a 97.2% turbidity reduction from dairy wastewater under optimized conditions [15]. Wang et al. investigated its application in low-concentration dye wastewater, where the decolorization rate in the cathodic chamber reached 70.6% within 150 min under specific operating conditions [16]. Despite its low energy consumption and high treatment efficiency, the 3D electro-Fenton process still faces technical limitations, including the high cost of electrode materials, poor dispersibility, and low long-term stability of treatment effects [17,18]. For instance, noble metal-based electrodes, although effective, are cost-prohibitive for large-scale applications. In addition, some composite materials tend to agglomerate during operation, leading to decreased dispersibility and impaired system stability.

In this study, biochemical sludge was utilized as a precursor to prepare iron-doped mesoporous nano-sludge biochar through a combined iron salt activation and mechanical ball milling approach. The resulting biochar was applied as a particle electrode in a 3D electro-Fenton system, integrating iron-doped biochar, a 3D electric field, and Fenton chemistry for the synergistic degradation of brewing wastewater. The prepared material is expected to possess a mesoporous structure, nanoscale particle size, and high specific surface area, thereby offering abundant active sites for COD degradation. Multivalent iron ions were introduced to establish a Fe^2+^/Fe^3+^ redox cycle and a stable Fe-O-Si framework, aiming to improve oxidation efficiency and catalytic durability. This design is anticipated to enhance the electro-Fenton performance and improve the degradation efficacy of COD in brewing wastewater.

## 2. Experimental Section

### 2.1. Main Experimental Raw Materials, Reagents, and Equipment

Raw materials and reagents: Biochemical sludge was obtained from a sewage treatment facility located in Renhuai City, , China. Brewing wastewater was collected from a sauce-flavor liquor brewery in the same region. Ferric chloride was purchased from Sinopharm Chemical Reagent Co., Ltd. (Beijing, China), sulfuric acid from Chongqing Chuandong Chemical Group Co., Ltd. (Chongqing, China), and sodium hydroxide from Tianjin Kemiou Chemical Reagent Co., Ltd. (Tianjin, China).

Equipment: The experimental apparatus included a planetary ball mill (QM-3SP04, Nanjing Chishun Technology Development Co., Ltd.)(Nanjing, China), an electric blast drying oven (DHG-9140A, Shanghai Jinghong Experimental Equipment Co., Ltd.) (Shanghai, China), a tube furnace (QTP-1200×60(LL), Hefei Kejing Materials Technology Co., Ltd.) (Hefei, China), a DC regulated power supply (ST-155, Suzhou Shutai Industrial Technology Co., Ltd.) (Suzhou, China), a magnetic stirrer (LC-MSB-HD, Shanghai Lichen Bangxi Technology Co., Ltd.) (Shanghai, China), a pH meter (PSH-3C, Shanghai Yidian Scientific Instruments Co., Ltd.) (Shanghai, China), and a COD digester (COD-HX12, Hangzhou Qiwei Instruments Co., Ltd.) (Hangzhou, China).

### 2.2. Experimental Methods

**Figure 1:**
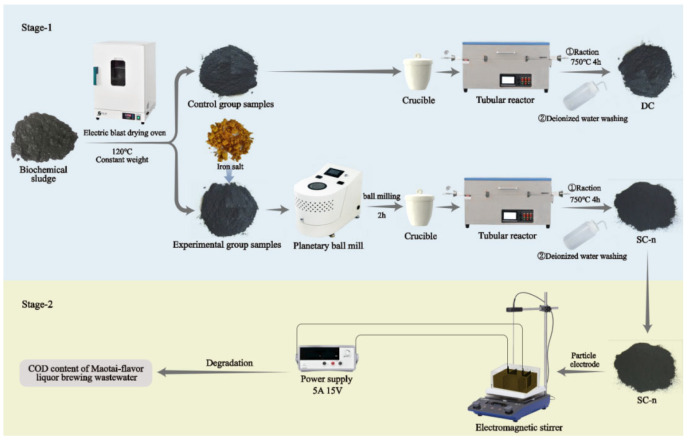
The schematic diagram of the experimental process.

The experiment comprised two distinct stages: material preparation and subsequent application. A schematic representation of the overall experimental procedure is provided in Figure 1.

The detailed experimental steps are as follows:

Stage 1: Preparation of Biochar Materials

A specified amount of biochemical sludge was dried to a constant weight at 105 °C in an electric blast drying oven. The dried sludge was mixed with ferric chloride (as the iron salt) at mass ratios of 5%, 10%, 15%, and 20%. The mixture was subjected to ball milling for 2 h using a planetary ball mill and subsequently dried again at 105 °C. The dried mixture was transferred to a crucible and placed in a tube furnace. Under a nitrogen atmosphere, the temperature was increased to 750 °C at a rate of 5 °C/min and maintained for 4 h. After natural cooling, the product was washed with deionized water until neutral and dried at 50 °C for 24 h to obtain the experimental samples. Control samples were prepared using the same procedure, excluding the iron salt activation and mechanical ball milling steps. The experimental samples were labeled as SC-n (where n represents the mass ratio of iron to dried biochemical sludge, i.e., 0.05, 0.1, 0.15, and 0.2), and the control sample was labeled as DC.

Stage 2: Degradation of Brewing Wastewater

The SC-n sample with the largest specific surface area was selected as the particle electrode to construct a 3D electro-Fenton system for brewing wastewater degradation. The main equipment of this system includes a DC regulated power supply, a magnetic stirrer, an electrolytic cell, electrode plates, and a pH meter. Among them, the voltage and current are adjusted via the rotary knob of the DC regulated power supply to control the current density passing through the electrode plates; the magnetic stirrer ensures the stable suspension of particle electrodes during the electrolysis process and provides a uniform electrolyte environment. The anode and cathode are made of ruthenium-iridium coated titanium plate and graphite plate, respectively, with dimensions (length × width × thickness) of 10 × 5 × 0.1 cm each.

First, place the electrolytic cell on the magnetic stirrer, then add a stir bar, particle electrodes, and brewing wastewater, and adjust the rotation speed of the magnetic stirrer to 1100 rpm. Fix the electrode plates with an iron stand and insert them into the electrolytic cell. Turn on the power and the magnetic stirrer to promote the uniform mixing of particle electrodes and brewing wastewater in the electrolytic cell. The reaction system operates at room temperature. Before the reaction, 1 M NaOH and H_2_SO_4_ are used to adjust the pH of the solution, which is measured with a pH meter.

A single-factor experiment was first conducted to examine the effects of four variables (particle electrode dosage, current density, pH, and electrolysis time) on the COD content of the wastewater. Based on the results of the single-factor experiment, the electrolysis time was fixed, and a response surface experiment was designed using the Box–Behnken method (Table 1) to optimize the brewing wastewater degradation process. In this experiment, the particle electrode dosage (A), current density (B), and pH (C) were set as independent variables, with the COD degradation rate of the wastewater as the response variable.

### 2.3. Analytical and Characterization Methods

The crystal structures and elemental valence states of the samples were examined using an X-ray diffractometer (X’Pert Powder Ⅲ, PANalytical B.V., Almelo, The Netherlands) and an X-ray photoelectron spectrometer (XPS, Thermo SCIENTIFIC ESCALAB 250Xi, Westborough, MA, USA), respectively.

The degree of carbon graphitization was assessed by Raman spectrometer (RM2000, Renishaw plc, Wotton-under-Edge, UK). Fourier transform infrared (FT-IR) spectroscopy (Nicolet-IS10, Thermo Fisher Scientific Inc., Westborough, MA, USA) was employed to characterize the functional group structures.

Sample morphologies and particle sizes were observed using a tungsten filament scanning electron microscope (SEM, SIGMA-500, Carl Zeiss AG, Oberkochen, Germany). Particle size distributions were further determined with a Zeta potential and particle size analyzer (Zetasizer Nano S90, Malvern Panalytical Ltd., Malvern, UK) and a laser particle size analyzer (Mastersizer 2000, Malvern Panalytical Ltd., Malvern, UK).

The specific surface areas and pore size distributions were analyzed using a surface area and porosity analyzer (NOVA1000E, Quantachrome Instruments, Boca Raton, FL, USA).

The COD of the brewing wastewater was measured in accordance with the Chinese national standard HJ828-2017 [19].

The electrochemical performance of the samples was tested using a CHI760E electrochemical workstation. First, 0.05 g of the SC-n material and 0.025 g of polytetrafluoroethylene (PTFE) binder were weighed, added to 2.5 mL of absolute ethanol for dispersion, and stirred thoroughly to form a homogeneous slurry. Subsequently, the obtained slurry was uniformly coated on the surface of a nickel foam (Ni) substrate, which served as the electrode current collector. After coating, the electrode was placed in an oven at 60 °C for 15 min to remove the solvent and fix the active material, finally obtaining the SC-n biochar electrode sheet. A three-electrode system was adopted, where the SC-n electrode acted as the working electrode, a platinum sheet as the counter electrode, and Ag/AgCl as the reference electrode; the electrolyte was a 0.5 M Na_2_SO_4_ solution. Cyclic voltammetry (CV) tests were conducted within a potential range of −0.4 V to 0.4 V (vs. Ag/AgCl) at a scan rate of 30 mV·s^−1^, aiming to evaluate the cyclic catalytic ability of the prepared catalyst for Fe^2+^/Fe^3+^ in the 0.5 M Na_2_SO_4_ solution. The test conditions for the control sample DC were consistent with those for the experimental sample SC-n.

## 3. Results and Discussions

### 3.1. Preparation of Biochar Materials

**Figure 2:**
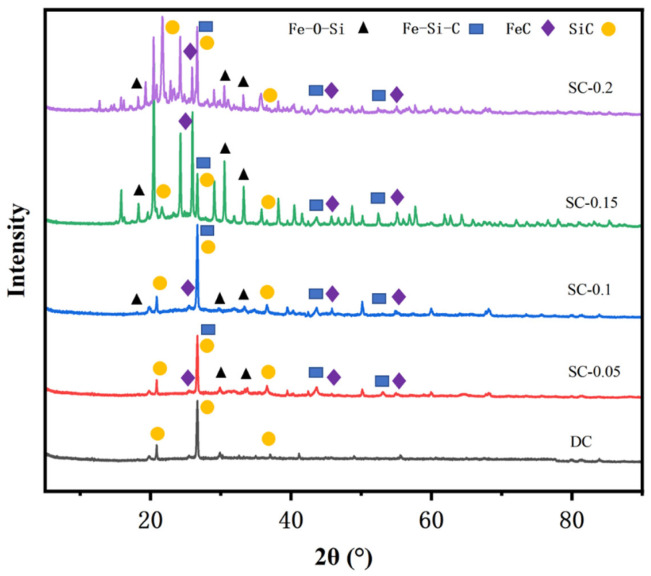
XRD Patterns of Samples.

Figure 2 shows the X-ray diffraction (XRD) patterns of the experimental and control samples. As observed from the figure, the DC sample (without iron salt activation) displays relatively distinct and sharp characteristic peaks, predominantly corresponding to the original mineral carbon crystal phase, indicating high crystallinity [20]. Diffraction peaks corresponding to SiC (silicon carbide), which matches the standard card PDF#42-1360, were observed at diffraction angles of 21°, 26°, and 36°. For the iron-doped activated samples, the number of diffraction peaks gradually increases with the iron doping amount. For example, the SC-0.05 sample shows only a few new peaks, whereas the SC-0.2 sample exhibits a more complex pattern with several overlapping peaks. This phenomenon indicates that the introduction of iron salt alters the crystal phase structure of biochar, while mechanical ball milling enhances the interaction between iron and the biochar matrix, leading to the formation of new phases such as iron-containing oxides and iron-oxygen-silicon compounds [21,22]. For the high iron-doped samples, diffraction peaks corresponding to Fe-O-Si (matching the standard card PDF#17-0548) were observed at diffraction angles of 19°, 27°, and 35°; peaks corresponding to Fe-Si-C (matching the standard card PDF#18-0651) were observed too. The characteristic peaks of the low-doping samples (SC-0.05, SC-0.1) shift slightly and are accompanied by the emergence of numerous weak peaks, suggesting that a small amount of iron is embedded in the pores or on the surface of biochar in a dispersed state (e.g., Fe^3+^) without forming substantial crystalline phases. In contrast, the medium-high doping samples (SC-0.15, SC-0.2) show a significant increase in the number and intensity of new peaks, which implies that the synergistic effect of higher iron salt concentration and mechanical milling promotes the formation of iron crystalline phases and enhances the orderliness of crystal grains. The low-doping samples exhibit good iron dispersibility but a limited number of crystalline phases, which may result in insufficient catalytic active sites. Although the high-doping samples contain abundant crystalline phases, iron agglomeration could reduce the number of effective active sites. The optimal doping sample (e.g., SC-0.15) achieves a balance between “dispersed iron and an appropriate amount of crystalline phases”, which enhances the COD degradation efficiency of brewing wastewater.

**Figure 3:**
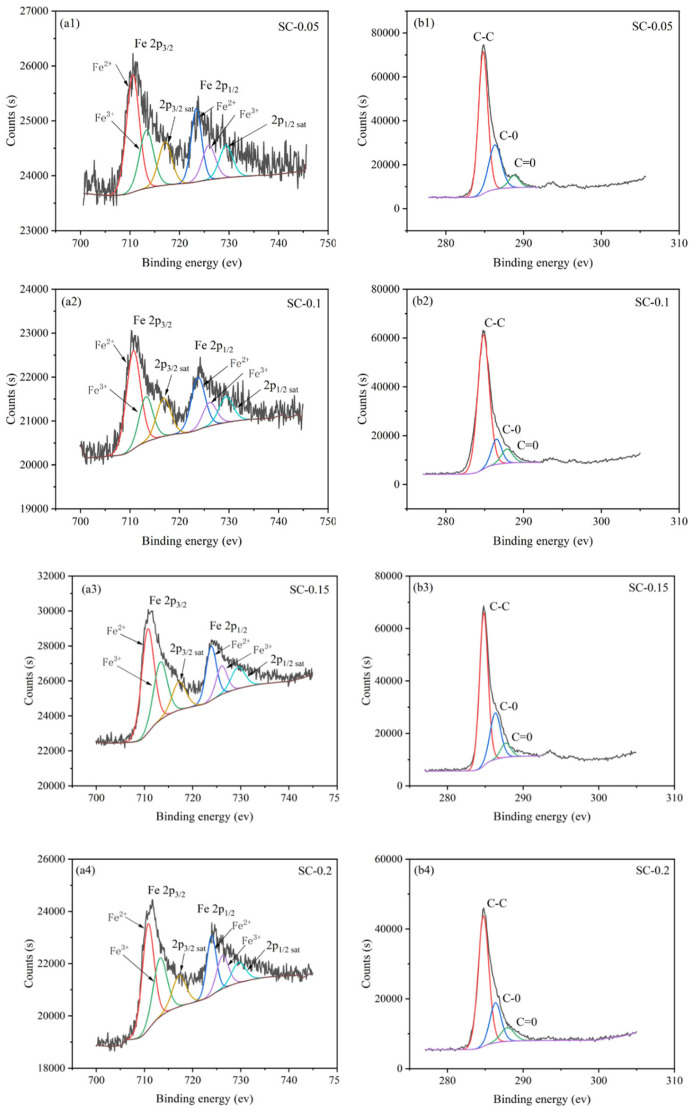
High-Resolution XPS Spectra of Samples (Fe: (**a1**–**a4**); C: (**b1**–**b4**)).

To further explore the doping form of iron salt and its impact on the structure of the prepared biochar material, high-resolution XPS peak fitting analysis of Fe 2p and C 1s was performed, with the results shown in Figure 3. As shown in the Fe 2p high-resolution spectra (a1–a4), all iron-doped samples (SC-0.05, SC-0.1, SC-0.15, and SC-0.2) exhibit characteristic peaks at approximately 714.4 eV (Fe 2p_3_/_2_) and 724.5 eV (Fe 2p_1_/_2_). As the iron salt addition increases, the intensity of these peaks progressively intensifies, indicating successful doping of iron into the biochar and an increase in the doping amount with higher iron salt concentration. In conjunction with related studies [23,24,25], these characteristic peaks confirm that iron exists in biochar in multiple chemical states (e.g., Fe^2+^ and Fe^3+^), providing active sites for the material’s participate in the 3D electro-Fenton reaction [26,27].

In the C 1s high-resolution spectra (b1–b4), all samples display characteristic peaks for C–C bonds at approximately 284.6 eV, C–O bonds at approximately 286.3 eV, and C=O bonds at approximately 287.9 eV. When comparing samples with different iron salt concentrations, the relative intensity of the C–O bond peaks tends to increase with the rise in iron doping. This suggests that during the iron salt activation process, iron species interact with the oxygen-containing functional groups on the biochar surface, altering the distribution of these groups within the carbon structure. This may influence the hydrophilicity/hydrophobicity and electron transfer performance of biochar, thereby positively affecting the subsequent 3D electro-Fenton degradation of organic wastewater from sauce-flavor liquor brewing—for example, enhancing the generation and mass transfer efficiency of active species such as OH (Further relevant data can be found in the Supplementary Data File).

**Figure 4:**
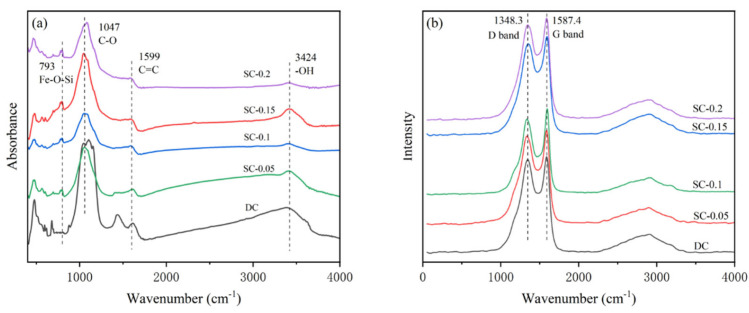
FT-IR Spectrum (**a**), Raman Spectrum (**b**).

Figure 4a presents the FT-IR spectra of the samples. As shown, with increasing iron doping, the vibration peaks associated with iron-related chemical bonds become more pronounced. An absorption peak corresponding to the Fe-O-Si bond appears around 793 cm^−1^ [28,29], and its intensity increases with higher iron doping levels. The intensity of the absorption peaks for surface functional groups (such as hydroxyl and carboxyl groups) also varies to some extent, suggesting that iron doping may influence the distribution and quantity of surface functional groups on biochar [30]. This effect is likely due to the complexation reaction between iron ions and functional groups, resulting in shifts, splits, or intensity changes of the corresponding peaks. Given the presence of silicon in the sludge, iron reacts with silicon and oxygen to form a Fe-O-Si structure during the iron salt activation and high-temperature calcination processes [31,32]. The appearance of this peak not only confirms the chemical bonding between iron and silicon in the sludge but also indicates an improvement in material stability, which further impacts its performance in the 3D electro-Fenton system.

Figure 4b shows the Raman spectra of the samples which represent the degree of disordered carbon and graphitized carbon, respectively. The figures reveal two broad peaks at approximately 1348.8 and 1578.4 cm^−1^, corresponding to amorphous and graphitized carbon [33,34]. The I_D_/I_G_ calculated as 0.969, 0.942, 0.929, 0.882, and 0.934, respectively, decrease with increasing iron doping, reaching a minimum of 0.882 for the SC-0.15 sample. However, the I_D_/I_G_ ratio increases again when the iron doping level is further increased (SC-0.2 sample). This trend suggests that the degree of graphitization improves with increasing iron doping. Iron salt doping serves as nucleation sites for carbon graphitization, promoting the ordered stacking of aromatic carbon structures, which enhances the material’s electrical conductivity. However, excessive iron doping may lead to the agglomeration of iron species, disrupting the continuous formation of ordered graphitized structures and introducing new structural defects. From the perspective of electro-Fenton degradation, moderate graphitization enhances electrical conductivity, accelerating electron transfer in the 3D electrode system and facilitating the generation of active oxidative species such as ·OH. In contrast, structural defects caused by excessive iron doping reduce electron transfer efficiency and may hinder the exposure of active sites due to iron agglomeration, weakening the degradation efficiency of organic wastewater from sauce-flavor liquor brewing. These findings provide a basis for optimizing iron salt doping levels and balancing the graphitization degree with the degradation activity of the material. (Further relevant data can be found in the Supplementary Data File).

**Figure 5:**
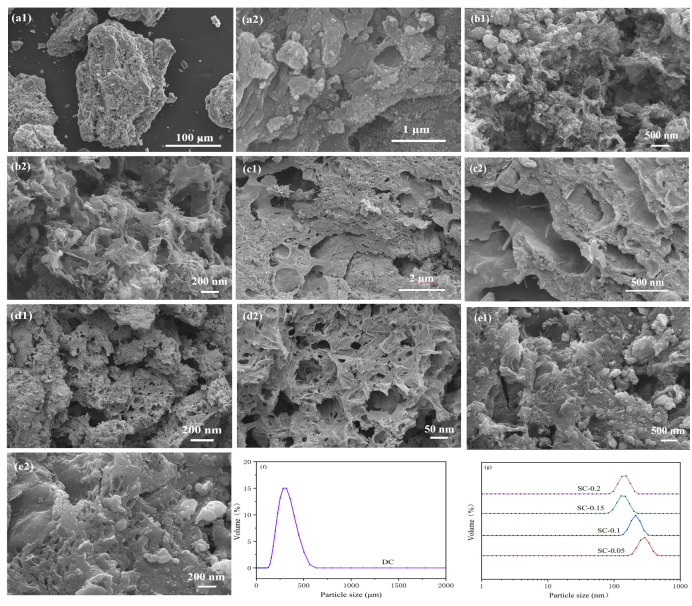
SEM Images (DC: (**a1**,**a2**); SC-0.05: (**b1**,**b2**); SC-0.1: (**c1**,**c2**); SC-0.15: (**d1**,**d2**); SC-0.2: (**e1**,**e2**)) and Particle Size Distributions (**f**,**g**) of Samples.

Figure 5 presents the scanning electron microscopy (SEM) images and particle size distribution diagrams of the samples. As shown in the figures, with increasing iron doping, the samples progressively develop porous structures: the DC sample exhibits a relatively dense, blocky morphology, while the SC series samples show an increase in pore numbers and a broader range of pore sizes. This indicates that iron doping has a pore-forming effect—during the iron salt activation process, the introduction of iron salts may induce the reconstruction of the internal structure of sludge-derived biochar, facilitating pore formation. Additionally, mechanical ball milling contributes to particle refinement. As observed in the particle size distribution diagrams (f and g), after mechanical ball milling, the particle size of the samples decreases significantly. Compared to the DC sample, the SC series samples exhibit a particle size distribution more concentrated in the nanoscale range, achieving a nanomaterial effect. This mesoporous nanostructure increases the specific surface area of biochar, providing more active sites for the 3D electro-Fenton reaction and enhancing the catalytic generation of active species such as ·OH. Moreover, the excellent dispersibility and mass transfer advantages of nanoscale particles improve the mass transfer efficiency within the electro-Fenton system, thereby enhancing the degradation efficiency of organic matter in brewing wastewater.

**Figure 6:**
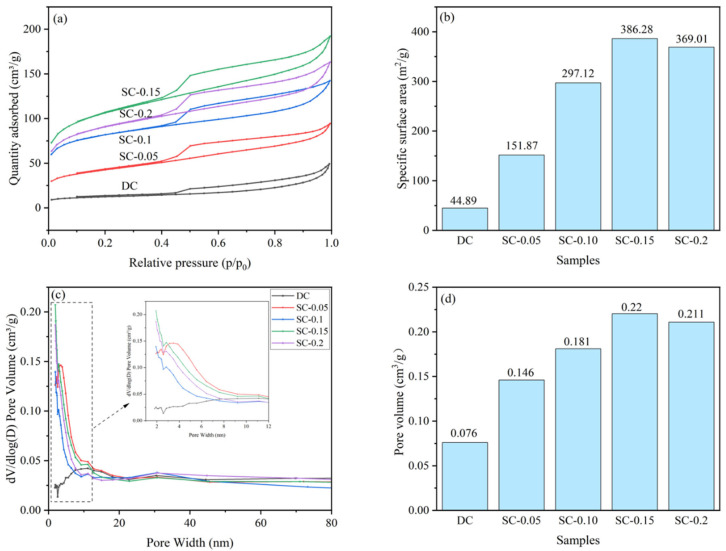
N_2_ Adsorption–Desorption Isotherms (**a**), Specific Surface Area (**b**), Pore Size Distribution (**c**), and Pore Volume (**d**) of Samples.

Figure 6 displays the N_2_ adsorption–desorption isotherms, specific surface areas, pore size distributions, and pore volumes of the samples. As can be seen from the figure, the N_2_ adsorption–desorption isotherms of all samples exhibit obvious hysteresis loops in the medium-high pressure region (p/p_0_ > 0.4), indicating enhanced mesoporosity. As the iron doping level increases from SC-0.05 to SC-0.15, a progressive rise in adsorption capacity is observed. Specifically, the specific surface area increases markedly from 44.89 to 386.28 m^2^/g, while the pore volume expands from 0.076 to 0.22 cm^3^/g (SC-0.15). Concurrently, the pore size distribution reveals a higher proportion of mesopores.

These results suggest that both particle size reduction and pore-forming effects contribute to the increase in surface area [35,36,37]. The synergistic activation by iron salts and mechanical ball milling not only promotes the development and expansion of internal porosity in sludge-derived biochar through iron species but also facilitates particle refinement, ultimately constructing a well-developed mesoporous network. This hierarchical porous structure provides abundant reactive interfaces and efficient mass transfer channels, which are beneficial for the 3D electro-Fenton degradation of organic wastewater generated from sauce-flavor liquor production, enhancing pollutant adsorption and catalytic oxidation processes.

### 3.2. Degradation of Brewing Wastewater

**Figure 7:**
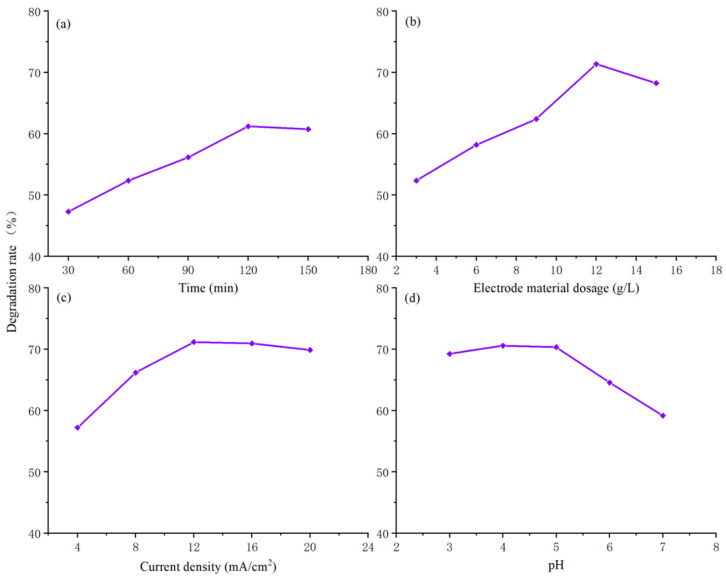
COD Degradation Rates of Brewing Wastewater Under Different Factors ((**a**): Time; (**b**): Particle Electrode Dosage; (**c**): Current Density; (**d**): pH).

Based on the comprehensive characterization results, the SC-0.15 sample exhibits a smaller particle size and a larger specific surface area, which enhances its potential for COD degradation in brewing wastewater. This sample was used as the particle electrode in a 3D electro-Fenton degradation system, and single-factor experiments were performed to investigate the influence of four factors—electrolysis time, particle electrode dosage, current density, and pH—on the COD degradation rate of brewing wastewater. The results are shown in Figure 7.

Figure 7a illustrates the experimental data on the effect of degradation time on the COD degradation rate of brewing wastewater. The experimental conditions were as follows: particle electrode dosage was 9 g/L, current density was 16 mA/cm^2^, and pH of the electrolytic solution was 4. The degradation times were 30, 60, 90, 120, and 150 min. As shown, the degradation rate increases with the extension of degradation time, reaching a maximum at 120 min, after which the rate of increase slows. This can be attributed to the continuous generation of active oxidative species, such as ·OH, during the early stage of the reaction, which rapidly react with organic pollutants in the brewing wastewater, driving an increase in the COD degradation rate. After 120 min, the easily oxidizable pollutants are mostly decomposed, and the proportion of refractory components increases. As a result, the generation and consumption of active species tend to balance, leading to a slowdown in the degradation rate.

Figure 7b shows the effect of particle electrode dosage on the COD degradation rate. The experimental conditions were degradation time of 120 min, current density of 16 mA/cm^2^, and pH of the electrolytic solution was 4. The particle electrode dosages were 3, 6, 9, 12, and 15 g/L. The data reveal that as the particle electrode dosage increases, the degradation rate rises rapidly, reaching a peak at 12 g/L, after which it decreases significantly. This is because an optimal increase in particle electrode dosage provides more catalytic active sites, promoting the decomposition of H_2_O_2_ to generate ·OH and enhancing oxidation efficiency. However, excessive electrode dosage leads to short-circuiting and shielding effects, and the electrodes settle more easily, reducing current efficiency and inhibiting ·OH generation, which ultimately causes a decline in the degradation rate.

Figure 7c presents the effect of current density on the COD degradation rate. The experimental conditions were degradation time of 120 min, particle electrode dosage of 12 g/L, and pH of the electrolytic solution was 4. The current densities were 4, 8, 12, 16, and 20 mA/cm^2^. As observed, the degradation rate increases gradually with the current density, but the rate of increase slows after the current density reaches 12 mA/cm^2^. This is due to the fact that increased current density enhances electron transfer on the electrode surface, promoting the reduction of O_2_ to generate H_2_O_2_ and accelerating the Fe^3+^/Fe^2+^ cycle, which increases the production of ·OH. However, when the current density exceeds 12 mA/cm^2^, side reactions, such as hydrogen and oxygen evolution, are intensified, consuming both electrical energy and active species. Additionally, excessively high current densities cause polarization on the electrode surface, inhibiting effective reactions and limiting further increases in the degradation rate.

Figure 7d shows the effect of pH value on the COD degradation rate. The experimental conditions were degradation time of 120 min, particle electrode dosage of 12 g/L, and current density of 12 mA/cm^2^. The pH values of the electrolytic solution were 3, 4, 5, 6, and 7. As seen in the figure, within the lower pH range, the degradation rate increases gradually, but when the pH exceeds 5, the rate decreases sharply. This is because in the electro-Fenton reaction, acidic conditions facilitate the Fe^3+^/Fe^2+^ cycle, maintaining catalytic activity, while H_2_O_2_ is stable and readily generates ·OH. However, when the pH exceeds 5, Fe^3+^ tends to hydrolyze, forming Fe(OH)_3_ precipitates that block the active sites on the electrode, hindering electron transfer and H_2_O_2_ decomposition. In alkaline conditions, ·OH is also easily deactivated by reacting with OH^−^, leading to a significant drop in degradation efficiency.

Based on the results of the preceding single-factor experiments, the response surface methodology (RSM) was employed to further optimize the brewing wastewater degradation process. The electrolysis time was held constant at 120 min, while particle electrode dosage (A), current density (B), and pH (C) were considered as independent variables. The COD degradation rate (Y) of brewing wastewater served as the response variable. Following the Box–Behnken experimental design principles, a total of 17 experimental points were selected for the response surface analysis, including 5 central-point experiments to estimate error. The results are presented in Table 2, and the analysis of variance (ANOVA) for the regression model is provided in Table 3.

A multiple linear regression analysis was conducted on the data in Table 2, resulting in the following quadratic polynomial regression equation that describes the relationship between the COD degradation rate (Y) of brewing wastewater and the independent variables particle electrode dosage (A), current density (B), and pH (C):

Y = 70.92 + 2.77A + 1.42B + 1.17C − 0.56AB − 1.53AC − 0.2BC − 4.22A^2^ − 2.89B^2^ − 0.44C^2^

As shown in Table 3, the model demonstrates an F-value of 26.2 and a *p*-value < 0.01, confirming its statistical significance. The *p*-value for the lack-of-fit term is 0.0740 (greater than 0.05), indicating that the lack of fit is not significant and suggesting that other factors have minimal impact on the experiment. This model is statistically valid and can reliably predict the COD degradation rate of brewing wastewater. Based on the magnitude of the F-values, the relative impact of each factor on the COD degradation rate of brewing wastewater is as follows: particle electrode dosage (A) > current density (B) > pH (C).

**Figure 8:**
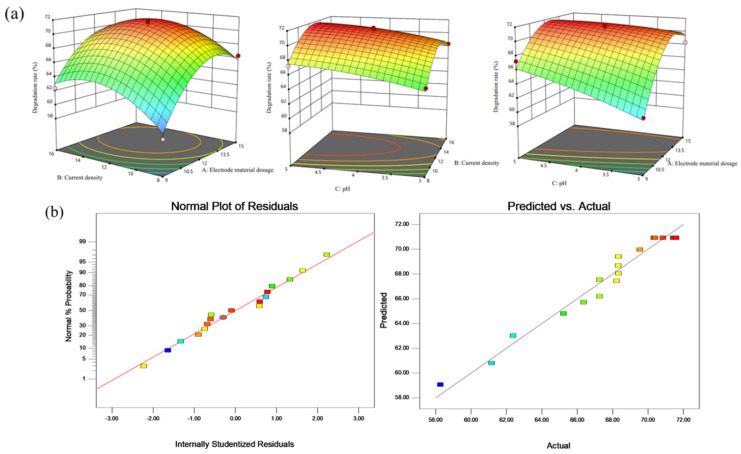
Response Surfaces and Contour Plots of Interaction Effects of Factors on COD Degradation Rate of Brewing. Wastewater (**a**); Normal Probability Plot of Residuals and Predicted vs. Actual Value Plot of Regression Model (**b**).

Figure 8 presents the response surfaces and contour plots illustrating the interaction effects of various factors on the COD degradation rate of brewing wastewater, along with the normal probability plot of residuals and the predicted vs. actual value plot of the regression model. The response surfaces and contour plots provide an intuitive representation of the interaction effects of the factors on the COD degradation rate. Steeper response surfaces and more elliptical contour plots indicate a stronger interaction between the factors. As seen in Figure 8a, the response surfaces of electrode dosage (A) and current density (B) exhibit relatively larger slopes, with more compact contour plots, signifying their considerable impact on the COD degradation rate. In contrast, the effect of pH (C) is less pronounced, which aligns with the ANOVA results presented in Table 3.

Figure 8b shows that both the normal probability plot of residuals and the predicted vs. actual value plot of the regression model are close to a straight line, with minimal dispersion. This suggests that the model’s predictions are accurate and reliable in real-world scenarios. Furthermore, the optimal process conditions determined through Design-Expert 11 software are as follows: particle electrode dosage of 12.4 g/L, current density of 12.79 mA/cm^2^, pH of 5, and degradation time of 120 min. Under these conditions, the theoretical COD degradation rate for brewing wastewater is 71.85%. For practical implementation, the parameters were adjusted to particle electrode dosage of 13 g/L, current density of 13 mA/cm^2^, pH of 5, and degradation time of 120 min. The actual degradation rate achieved was 71.12%, which is close to the predicted value, demonstrating that the model provides a good fit and can effectively guide the optimization of the COD degradation process for brewing wastewater.

**Figure 9:**
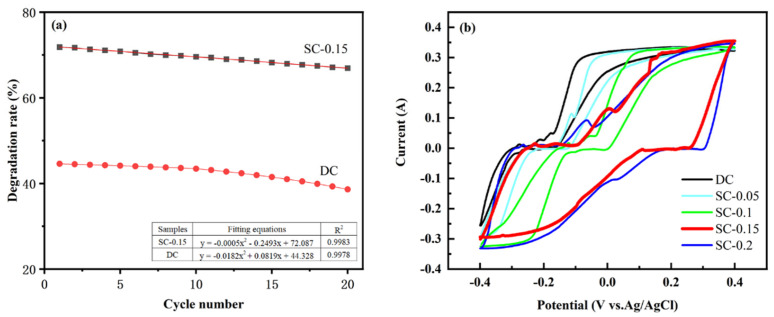
Cyclic Degradation Stability (**a**) and Current-potential redox (**b**) of SC-0.15 and DC Samples.

Under the optimal degradation conditions mentioned above, SC-0.15 and DC samples were used as particle electrodes to compare their degradation efficiency and cyclic stability. The results are shown in Figure 9a. As illustrated in the figure, the SC-0.15 sample, prepared using the iron salt activation-mechanical ball milling coupling technology, demonstrates a higher COD degradation rate and superior cyclic stability compared to the unmodified control sample. The initial degradation rate of SC-0.15 is significantly higher than that of DC, and its degradation rate decreases more slowly with increasing cycle numbers. Based on the fitting equations, the smaller absolute value of the quadratic coefficient for SC-0.15 indicates a slower rate of degradation attenuation. This phenomenon can be attributed to the synergistic effect of the mesoporous nanostructure formed by iron salt activation and ball milling. On the one hand, iron salt enhances the Fe^3+^/Fe^2+^ cycle in the electro-Fenton system, promoting the continuous and efficient generation of ·OH. On the other hand, the mesoporous structure provides abundant active sites and mass transfer channels, while mechanical ball milling improves the material’s dispersibility, preventing particle agglomeration and the loss of active sites during cycling. In contrast, the DC sample, which lacks iron doping and an ordered mesoporous structure, has fewer active sites and lower electron transfer efficiency, making it prone to structural collapse and catalytic activity degradation during cycling. These results highlight the advantages of the iron salt activation-mechanical ball milling coupling technology in enhancing the cyclic stability of biochar electrodes. Compared with other reported particle electrodes (such as bentonite (MBt), titanium/titanium dioxide nanotube/lead dioxide (Ti/TiO_2_ nanotube/PbO_2_), microbial cellulose/ferroferric oxide (microbial cellulose/Fe_3_O_4_), and ferroferric oxide/nitrogen-doped reduced graphene oxide (Fe_3_O_4_/N-rGO)), this method not only realizes the resource utilization of waste excess sludge but also enables the preparation of low-cost particle electrodes [16,17,18].

To further verify the interpretation of Figure 9a, the electrochemical performance of the prepared SC series samples and the control sample DC was tested, and the results are shown in Figure 9b. The cyclic voltammetry (CV) method was used to systematically evaluate the electrochemical behavior and catalytic performance of the prepared samples in this system. In Figure 9b, CV tests were performed on five different materials (DC, SC-0.05, SC-0.1, SC-0.15, and SC-0.2) at a scan rate of 30 mV·s^−1^ to assess their electrochemical performance and catalytic ability. The results show that the area under the CV curves of SC-0.15 and SC-0.2 samples is significantly larger than that of DC, SC-0.05, and SC-0.1, indicating that the former two have better electrochemical reaction activity. Meanwhile, it also reveals a trend that the electrochemical activity of the catalyst increases with the introduction and content increase of Fe. In particular, SC-0.15 exhibits an oxidation peak at 0.17 V (vs. Ag/AgCl) with a corresponding peak current of 0.31 mA, which is significantly higher than that of SC-0.2 (0.28 mA), suggesting that its electrode surface has a larger effective active area and faster electron transfer rate. In addition, the reduction peak of SC-0.15 appears at 0.26 V (vs. Ag/AgCl), and the potential difference (ΔEp) between its oxidation peak and reduction peak is 0.09 V, which is significantly lower than that of SC-0.2 (0.11 V), SC-0.1 (0.10 V), SC-0.05 (0.11 V), and DC (0.12 V). This result further indicates that the SC-0.15 catalyst has better reversibility in oxidation and reduction processes, faster electron transfer rate, and stronger reaction activity.

## 4. Conclusions

Iron-doped mesoporous nano-sludge biochar was successfully synthesized via a coupling process of iron salt activation and mechanical ball milling. The iron salt functions both as a dopant and a pore-forming agent. Iron is incorporated in a mixed divalent and trivalent state, providing abundant active sites for the electro-Fenton reaction. The average particle size of the material is 187 nm, significantly enhancing its specific surface area and surface properties. The material exhibits a reticular porous structure with a specific surface area of 386.28 m^2^/g, which is much higher than that of the iron-free sample. The mesoporous structure is confirmed by N_2_ adsorption–desorption isotherms and pore size distribution, optimizing the mass transfer effect and facilitating the interaction between active species and pollutants. Mechanical ball milling refines the particle size, reduces agglomeration, enhances surface defects, improves electron transfer performance, and thereby increases the cyclic stability of the material. When SC-0.15 is used as the particle electrode in a 3D electro-Fenton system, the COD degradation rate of brewing wastewater reaches 71.12% under optimal conditions, 61.1% higher than that of the unmodified material. After 30 cycles, the degradation capacity retention rate is 88.74%. This material, as a particle electrode in a 3D electro-Fenton system, demonstrates excellent organic matter degradation performance and holds strong potential for application in the treatment of organic wastewater from sauce-flavor liquor brewing.

## Figures and Tables

**Table 1:** Factors and Levels of Response Surface Experiment.

Factor	Coded Levels		
	−1	0	1
A. Particle electrode dosage (g/L)	9	12	15
B. Current density (mA/cm^2^)	12	16	20
C. pH	3	4	5

**Table 2:** Experimental Design and Results of Response Surface Methodology for COD Degradation Condition Optimization of Brewing Wastewater.

Experiment No.	A	B	C	COD Degradation Rate (%)
1	−1	−1	0	58.27
2	1	−1	0	66.36
3	−1	1	0	62.38
4	1	1	0	68.23
5	−1	0	−1	61.16
6	1	0	−1	68.33
7	−1	0	1	67.26
8	1	0	1	68.32
9	0	−1	−1	65.23
10	0	1	−1	68.33
11	0	−1	1	67.26
12	0	1	1	69.54
13	0	0	0	70.84
14	0	0	0	71.59
15	0	0	0	70.33
16	0	0	0	71.43
17	0	0	0	70.4

**Table 3:** Variance analysis of regression model.

Source	Sum of Squares	df	Mean Square	F-Value	*p*-Value	Significant
Model	217.90	9	24.21	26.20	0.0001	significant
A	61.44	1	61.44	66.47	<0.0001	
B	16.13	1	16.13	17.45	0.0041	
C	10.88	1	10.88	11.77	0.0110	
AB	1.25	1	1.25	1.36	0.2822	
AC	9.33	1	9.33	10.10	0.0155	
BC	0.17	1	0.17	0.18	0.6826	
A^2^	74.81	1	74.81	80.95	<0.0001	
B^2^	35.23	1	35.23	38.12	0.0005	
C^2^	0.80	1	0.80	0.86	0.3838	
Residual	6.47	7	0.92			
Lack of Fit	5.14	3	1.71	5.13	0.0740	not significant
Pure Error	1.33	4	0.33			
Cor Total	224.37	16				

## Data Availability

Data will be made available from the corresponding authors (Fuyong Wu: wufuyong@mtxy.edu.cn; Yi Xie: xieyi@mtxy.edu.cn) on reasonable request.

## Supplementary Materials

The following supporting information can be downloaded at: https://www.mdpi.com/article/10.3390/nano15191530/s1.
